# Temporomandibular Joint Disorders in Elderly Patients

**DOI:** 10.7759/cureus.76958

**Published:** 2025-01-05

**Authors:** El. Mehdi Jouhadi, Sara Rhattas, Ichraq Benazouz, Khalid Elboussiri

**Affiliations:** 1 Department of Fixed Prosthodontics, Faculty of Dental Medicine, Hassan II University of Casablanca, Casablanca, MAR; 2 Department of Biology and Basic Subjects, Faculty of Dental Medicine, Hassan II University of Casablanca, Casablanca, MAR

**Keywords:** aging, craniofacial pain, elderly patients, temporomandibular disorders, temporomandibular joint

## Abstract

The management of temporomandibular disorders (TMDs) in elderly patients can present a significant challenge for dentists due to its multifactorial etiology, aging-related changes that contribute to TMD, and the fragile psychological state of these patients. Despite the growing prevalence of TMD in the elderly population, the scientific literature provides limited information about effective management strategies for this group. Therefore, it is crucial for researchers and clinicians to focus on improving our understanding of TMD in the elderly population and to develop more effective treatment approaches. This article provides an overview of the prevalence, etiology, pathophysiology, and management of TMD in elderly patients, with a particular emphasis on the challenges associated with this population. By increasing our knowledge of TMD in the elderly, we can improve the quality of care provided to this vulnerable patient group.

## Introduction and background

Temporomandibular disorders (TMDs) are a subgroup of craniofacial pain disorders. TMDs are characterized by craniofacial pain involving the joint, masticatory muscles, or muscle innervations of the head and neck. TMDs are a major cause of non-dental pain in the orofacial region [1]. The American Academy of Orofacial Pain defines TMDs as an umbrella term, which covers a set of musculoskeletal and neuromuscular conditions involving the masticatory musculature, the temporomandibular joint (TMJ), and/or their associated structures [2]. TMDs affect up to 15-20% of adult patients, with a peak incidence at 20 to 40 years of age [3]. TMDs are the second most common chronic musculoskeletal condition after chronic low back pain [4]. Women tend to show more signs and symptoms of TMDs and to seek treatment more frequently as compared to men [5,6]. For individuals with TMDs, issues with the TMJ and the muscles surrounding it can cause a variety of symptoms, including muscle pain that radiates through the face, jaw, and/or neck; stiffness in the jaw and neck muscles; limited movement or locking of the jaw; painful clicking or popping in the jaw; and a change in the way the upper and lower teeth fit together [7]. Pain is the most characteristic feature in most TMD cases and the main reason patients seek treatment [5]. The etiology of the most common types of TMDs is complex and remains largely unresolved [4], and it seems that the etiology of TMDs is multifactorial and includes biological, environmental, social, emotional, and cognitive reasons [7]. TMDs in the elderly population have a number of peculiarities mainly in totally edentulous patients. Despite the relatively high prevalence of TMDs in the elderly population, there are few review articles that focus on this specific age group. TMDs in elderly patients present several particularities that need to be well understood and mastered by the dentist in order to treat these patients effectively.

The aim of this article is to provide a general overview of TMDs in the elderly population with regard to their prevalence, etiology, pathophysiology, and management.

## Review

Prevalence

The exact prevalence of TMDs in the general population varies greatly in the literature and ranges from 3% to 33%, which might be the consequence of considerable variations in diagnostic protocols [8]. Recently, a systematic review investigated the prevalence of TMDs among the general population, using diagnostic criteria recognized worldwide such as Research Diagnostic Criteria for Temporomandibular Disorders (RDC/TMD) and Diagnostic Criteria for Temporomandibular Disorders (DC/TMD), in order to summarize the data collected over the years for epidemiological purposes, has shown that adults/elderlies have a higher prevalence of TMJD (31.1%) than children/adolescents (11.3%). Some studies have examined the prevalence in the older population, with a reported prevalence of at least one symptom in this population of between 40% and 80% [9,10]. Numerous studies investigating the prevalence of TMDs among older adults have yielded varying results. While some research suggests that TMD prevalence in elderly populations remains relatively low, findings across studies lack consensus [11,12,13]. However, a study realized by Sampaio et al. (2017) showed a high prevalence of TMDs in the elderly, other studies [14] showed that there is a correlation between increasing age and increasing potential for the development of signs and symptoms of TMD, and other authors reported that the elderly can often suffer from TMDs but do not complain because they are more concerned with other overall health issues or more painful and debilitating diseases, leaving aside the symptoms related to the masticatory system [15]. Further studies of the prevalence of TMDs in the elderly population should be conducted since the previous correlated studies adopted different diagnostic criteria. Gender appears to be a significant factor in the prevalence of TMDs, with several studies reporting a higher incidence of TMDs in women compared to men [16,17,18]. This disparity is further supported by the impact of fluctuating estrogen levels throughout various life stages: elevated estrogen levels during pregnancy are linked to increased gingivitis, while reduced estrogen levels during menopause contribute to TMJ degeneration and alveolar bone loss [19]. However, some studies have found no significant association between gender and TMDs [20], suggesting that additional research is needed to fully understand the complex relationship between gender and TMDs.

Etiology

The etiology of the most common types of TMDs is complex and remains largely unresolved [4]. In general, TMDs have a multifactorial cause, with an interaction of systemic, psychosocial, genetic, trauma-related, hormonal, neurological, and anatomic or facial morphology factors [21]. In older subjects, several factors have been suggested as responsible for the development of TMDs. The elderly population is characterized by the phenomenon of senescence, which involves a variety of physiological changes associated with aging. These changes can have a significant impact on the TMJ and surrounding muscles, potentially contributing to the development of TMDs in elderly individuals. Numerous studies have investigated the relationship between age and TMJ changes, including Agerberg et al. (1989) [22], Manfredini et al. (2010) [23], and Alexiou et al. (2009) [24]. These studies have demonstrated that, similar to other joints in the body, the TMJ can degenerate with age [22,23,24], potentially leading to pathological changes that increase the risk of TMDs. Faulty vertical dimension is a common cause of muscular pain among complete denture wearers, due to the muscle hyperactivity, which resulted from the increased level of contraction. Budtz-Jorgensen et al. [25] and Togelberg and Kopp [26] stated that the loss of occlusal support was positively correlated with the severity of TMDs. There are several oral and dental factors that may be associated with TMD signs and symptoms, including posterior tooth loss, edentulism, and denture use. Loss of molar support has long been considered an important etiological factor for TMDs [27,28,29]. Other studies have concluded that among the elderly population, the severity of TMDs does not depend on the supporting zones of the dentition alone, and removable prostheses do not relieve the problem [30,31]. According to a study conducted by E. Dervis [32], no statistically significant correlations were found between signs and symptoms of TMDs and denture retention, stability, occlusal errors, freeway space, age of present denture, or number of sets of dentures. In another study [33], no robust association between prosthetic factors and TMDs was found. There are also psychological factors (anxiety and stress mainly), postural, traumatic, and parafunctional oral habits that can cause TMDs. Several psychosocial factors can not only predispose a person to TMD pain but also prolong it [34,35]. The biopsychosocial model, which involves a combination of biological, psychological, and social factors, is the most widely accepted theory to explain TMDs [36].

Physiopathology

Aging affects all the organs and tissues of the body, including the TMJ. Aging is a physiological process that is accompanied by a series of cellular, molecular, and structural changes that influence the properties and function of the tissues [37]. With aging or other pathological conditions, senescent cells accumulate in multiple tissues including joints, secreting a variety of pro-inflammatory cytokines, chemokines, and proteases, termed the senescence-associated secretory phenotype (SASP) [38]. Research conducted by Po-Jung Chen et al. [39] has documented aging-associated changes in the extracellular matrix and cells of the cartilage of the TMJ. The most important changes observed were decreased proteoglycan secretion, decreased cellularity, decreased cartilage thickness, increased fibrillation, and increased proteolytic activity. A recent systematic review conducted in 2020 by Yuanyuan Yin et al. aimed to better understand the neurobiological underpinnings of TMD-related pain. The review found evidence for the existence of both a peripheral and central neural basis for pain in TMDs and also revealed alterations in several cortical regions implicated in pain perception and pain modulation in TMDs [40].

Management of TMDs

To carefully manage elderly patients suffering from TMDs, the dentist should perform a careful medical interview. The doctor-patient relationship lies at the heart of healthcare, and patient trust is a fundamental aspect of that relationship, especially for older patients. Behavioral counseling should also be provided to patients, as the goal of behavioral counseling is to allow the patient to take "self-management" of their dysfunction (i.e., stress reduction, sleep hygiene, elimination of parafunctional habits). Treatment of TMDs can be conservative or surgical. Surgery is rarely required for the management of TMDs and is usually reserved for the correction of anatomic or disk problems. Surgical options include arthrocentesis, arthroscopy, and open joint surgery [7]. Conservative treatments consist of patient education, jaw exercises, massage, thermal therapy, dietary advice and nutrition, parafunctional behavior identification, monitoring, and avoidance [41].

Pharmacologic management

Effective pain management is a critical component of patient care, with recent systematic reviews indicating that analgesic use significantly enhances treatment efficacy by reducing both pain intensity and cytokine levels, thereby addressing inflammatory pathways [42]. This is achieved through both their analgesic and anti-inflammatory actions, and the efficacy is similar for both levels II and III. Nonsteroidal anti-inflammatory drugs (NSAIDs) aid in the management of TMDs through their anti-inflammatory and analgesic properties. NSAIDs have several adverse effects, the most important of which is their effect on the gastrointestinal (GI) tract. In addition, NSAIDs can interact with multiple medications and result in unwanted effects. For instance, NSAIDs may decrease lithium clearance, increase the serum concentration of lithium, and cause toxicity. Although it is not clear who is predisposed to this interaction, elderly patients are likely most susceptible [43]. Tricyclic antidepressants are used for the management of chronic TMD pain. Benzodiazepines are also used but are generally limited to two to four weeks in the initial phase of treatment [44]. Injection is a treatment option that encompasses various methods of administration. Infiltration, considered an invasive yet conservative and reversible technique, is commonly used. Local anesthetics and corticosteroids are the most frequently injected substances. Moreover, recent developments in regenerative medicine have introduced platelet-rich plasma (PRP) and hyaluronic acid (HA) as potential adjuncts in TMJ arthrocentesis. PRP, derived from the patient's blood, is rich in growth factors and cytokines that support tissue healing and regeneration. Hyaluronic acid, a natural component of synovial fluid, provides lubrication and anti-inflammatory effects, offering a promising approach to pain relief and joint health improvement [45]. The use of onabotulinum toxin (Botox) for TMDs and bruxism lacks conclusive evidence of its effectiveness. Furthermore, systematic reviews have reported several side effects, including muscle weakness, injection site tenderness, asymmetric smile, difficulty swallowing, and swelling [46,47,48]. 

Physical therapy

The primary goal of physical therapies, including ultrasound, heat, laser, electro-stimulation, relaxation exercises, stretching, and biofeedback, is to alleviate pain, enhance muscle strength, improve coordination, promote relaxation, and increase the range of motion. According to Melis et al. [49], low-level laser therapy (LLLT) appears to be more effective for treating TMJ disorders but less effective for managing masticatory muscle disorders. In addition, a meta-analysis demonstrated improved treatment outcomes when acupuncture was combined with magnetic therapy, a form of physical therapy. Moreover, acupuncture has gained recognition as a promising alternative therapy, particularly for patients who face limitations with traditional treatment options [50].

Prosthodontic treatment

Patients with TMDs should be managed carefully. Treatment options for elderly patients with TMDs may include occlusal rehabilitation, such as new complete dentures, occlusal splints, sliding plates, or central bearing. The efficacy of occlusal adjustments in preventing or managing TMDs remains uncertain and warrants further research [51].

New Complete Dentures

Elderly patients often wear complete dentures for extended periods, which can increase their risk of developing TMDs. Abdelnabi et al. [52] found that new dentures with corrected occlusion significantly improved clinical signs and symptoms of TMDs in complete denture wearers, including disc position. Therefore, new complete dentures may be a suitable treatment option for TMDs in this population.

Occlusal Splints

Prosthetic treatment with occlusal splints is recommended for both dentate and edentulous patients. The occlusal splint allows for an increase in the DVO, which results in a decrease in pressure in the joint space and on the disc. This reduced pressure allows the establishment of new muscle action patterns [53]. Splint therapy is regarded as an important aspect of TMD care, partially and completely edentulous patients present a special challenge because the lack of stability of the complete or partial denture may also complicate care [54].

In partially edentulous patients, Lim demonstrated that the best alternative is to incorporate a removable partial denture (RPD) into the splint. In complete edentulous patients, Mariana et al. showed that superior repositioning splints (SRSs), followed by the fabrication of new prostheses, successfully treated TMDs and maintained asymptomatic conditions over a 10-year follow-up. This underscores the effectiveness of SRS in managing TMDs. These approaches highlight the importance of utilizing splint therapy before fabricating final prostheses, ensuring improved long-term outcomes for edentulous patients with TMDs [54,55,56].

Sliding Plates

Complete dentures with posterior sliding plates should be considered an interim prosthesis to be used before the preparation of a definitive prosthesis. Sliding plates are smooth surfaces fitted to the occlusal aspect of the posterior teeth of complete maxillary and mandibular dentures and have the same function as the smooth surfaces of an occlusal splint [57]. Another study has demonstrated that sliding plates may offer significant benefits to completely edentulous patients experiencing painful symptoms, with the goal of reestablishing the occlusion vertical dimension and muscular function promoting, the freedom of mandibular movements, and allowing a more physiological position of the mandible [58]. 

## Conclusions

TMDs in the elderly represent several particularities, primarily related to physiopathology. Symptoms of TMDs in the elderly population present simultaneously with biochemical and physiological alterations associated with aging. Management of TMDs may be a challenge in elderly patients for a dentist, due to its multifactorial etiology, aging changes that lead to TMD, and the fragile psychological state of those patients. The doctor-patient relationship lies at the heart of health care, and patient trust is a fundamental aspect of that relationship, especially for older patients. Recently, the literature reports little information about this population, authors must be more interested in TMDs in the elderly in order to be treated efficiently.
